# Cavernous Nerve Injury Resulted Erectile Dysfunction and Regeneration

**DOI:** 10.1155/2021/5353785

**Published:** 2021-12-21

**Authors:** Nan Jiang, Cheng Wu, Xunrong Zhou, Guanghua Zhai, Jian Wu

**Affiliations:** ^1^Department of Urology, People's Hospital of Dongtai City, Dongtai, Jiangsu, China; ^2^Department of Clinical Laboratory, The Affiliated Suzhou Hospital of Nanjing Medical University, Suzhou Municipal Hospital, Gusu School, Nanjing Medical University, 242 Guangji Road, Suzhou 215008, Jiangsu, China

## Abstract

Erectile dysfunction (ED) is an important cause of reduced quality of life for men and their partners. Recent studies have found that cavernous nerve injury (CNI) during prostate cancer surgery and other pelvic surgery results in medically induced CNIED in more than 80% of patients. The efficacy of first- and second-line treatment options for ED is poor. A great deal of research has been devoted to exploring new methods of neuroprotection and nerve regeneration to save erectile function in patients with CNIED, especially in patients with cavernous nerve injury after prostate cancer surgery. In addition, such as neuromodulatory proteins, proimmune ligands, gene therapy, stem cell therapy, and the current cutting-edge low-energy shock wave therapy have shown advantages in basic research and limited clinical studies. In the context of today's modern medicine, these new therapeutic techniques are expected to be new tools in the treatment of cavernous nerve injury erectile dysfunction. This article presents the main causes, mechanisms, and treatment of cavernous nerve injury erectile dysfunction and combines them with new treatment strategies.

## 1. Introduction

Erectile dysfunction (ED) is a male sexual disorder in which there is repeated or persistent difficulty achieving or maintaining an erection with a prevalence of 30-64% in men aged 40 to 79 years. Erectile dysfunction has a variety of causes, and with the widespread introduction of pelvic surgery in recent years, a type of neurologically injurious erectile dysfunction is becoming a focus of urological research, namely, cavernous nerve injury erectile dysfunction (CNIED). A global multicentre study conducted by the European Male Ageing Study (EMAS) has shown that CNIED accounts for over 14% of the total number of patients with ED [1, 2].

### 1.1. Anatomical Physiology of the Cavernous Nerve

The cavernous nerve (CN) is the main autonomic nerve regulating penile erection and is involved in the voiding reflex by innervating the urethral transverse muscle on the anterior aspect of the prostate through branches. Han et al. first probed the course of the CN by evoking electrical stimulation during RP and detailed the male fetal and neonatal cavernous innervation pathways, demonstrating that disruptions in the conduction pathways of the erectile reflex arc are a direct contributor to ED [3]. The CN originates primarily from the pelvic plexus and contains sympathetic nerves that originate at the T11-L2 level and parasympathetic nerves that originate at the S2-S4 level. The two nerves branch out of the pelvic plexus and intersect with each other to form the CN, which travels lateral to the prostatic fascia and joins with several arteriovenous branches to form a very thin neurovascular bundle, called the neurovascular bundle (NVB), which is covered by the prostatic envelope and travels up the prostate. The cavernous nerve enters the corpus cavernosum of the penis via the tip of the prostate and travels along the sides of the membranous urethra, where its nerve fibres innervate the penis by releasing neurotransmitters. In response to various stimuli, sexual impulses from the cerebral cortex or peripheral penile nerves excite the parasympathetic fibres of the CN, and a combination of neurotransmitters, neuronal NOS (nNOS) and endothelial NOS (eNOS) in the vascular and sinusoidal endothelium, is released in the nerve endings to initiate penile erection.

### 1.2. Etiology and Epidemiology

Medically induced injury is the most common cause of CN injury, with radical prostatectomy (RP) being the cause of increasing interest in recent years. Prostate cancer (PCa), the second most prevalent malignancy in men, has seen recent advances in diagnostic techniques, yet RP remains the preferred strategy for early treatment [4]. Due to the special anatomy of CN and the fact that the lateral prostatic space is not well defined during surgery, it is easy to damage the NVB between the two layers of fascia, and even with bilateral preservation of the nerve and minimally invasive RP, some degree of CN injury (CNI) or nerve loss is inevitable [2]. Despite the recent promotion of CN preservation and the continued implementation of robotic-assisted lumpectomy RP, there is a precipitous decrease in erectile function (EF) after RP, including a reduction or loss of nocturnal spontaneous erection and a reduced erectile response to stimulation. The incidence of ED was estimated to be as high as 84.6% in patients who had no CN on either side and remained as high as 24%-50% after unilateral or bilateral CN [5]. By 12 months after RP, EF had not recovered in over 70% of patients [6], and in some patients, complete erectile dysfunction persisted for more than 18 months [7]. The clinical gold standard for BPH is transurethral resection of the prostate (TURP), with 4-35% of patients presenting with postoperative ED, some due to intraoperative cavernous nerve injury [8]. Other medical CN injuries, including radical surgery for pelvic tumours such as bladder cancer, colorectal cancer, and radiotherapy for prostate cancer, are direct or indirect acute and chronic CN injuries caused by intraoperative pulling, clamping, dissection, freezing, electrocautery, excision, and irradiation.

Nonmedical injuries leading to cavernous nerve injury erectile dysfunction are mainly caused by pelvic trauma, including pelvic nerve injury and posterior urethral injury-causing CN disuse, in which the proportion of patients with deep abdominal nerve injury complicating ED is as high as 95.12% [9]. Injuries to the posterior urethra also tend to cause damage to the CN of the membranous urethral travel leading to different degrees of ED. Other nerve injuries in erectile dysfunction are mainly lesions of the upstream regulatory center or spinal cord, or lesion-type injuries represented by CNIED due to diabetes mellitus [10].

### 1.3. The Mechanism of Erectile Dysfunction

It is generally accepted that the neurotransmitter nitric oxide (NO) and the constitutive NO synthase (NOS) isoforms are important mediators of penile erection [11]. Combinatorial NOS are phosphorylated and activated to produce NO, which diffuses into smooth muscle tissue to facilitate the conversion of guanosine 5t-triphosphate (GTP) to the second messenger cyclic guanosine monophosphate (cGMP). Activation of protein kinases (PKG) by cGMP accelerates protein phosphorylation and decreases calcium concentration, which leads to cavernous smooth muscle diastole, completes the process of cavernous tissue engorgement to penile erection, and participates in the subsequent maintenance of the erectile state. In contrast, it takes six months to two years to restore erectile function to the penis after RP by combined prostilol and PDE5i injections and adjuncts such as vacuum negative pressure, during which the lack of natural erection leads to penile hypoxia, and altered neurovascular mechanisms were previously thought to be the main cause of CNIED [12, 13]. In addition to ischaemic injury to the corpus cavernosum, several studies have shown that apoptosis and fibrosis of smooth muscle cells within the corpus cavernosum tissue caused by CN injury are more clinically relevant to ED after RP. Compared to the preoperative and early postoperative periods (<2 months), both elastic fibres and smooth muscle cells in the cavernous tissue were significantly reduced and collagen content was significantly increased in the late postoperative period (>12 months) [14]. After CN injury, sphincter integrity is disrupted, nNOS-positive nerve density is reduced, continuity of the CN reflex pathway is reduced or lost, and NO expression, a signaling molecule that mediates relaxation of cavernous smooth muscle and maintains penile erection, is severely downregulated [15].

Antifibrotic compounds within smooth muscle cells inducible nitric oxide synthase (iNOS) are downregulated, transforming growth factor beta 1 (TGF*β*1) is upregulated, and prostaglandin E1 (PGE1) and cyclic adenosine monophosphate (cAMP) are inhibited. TGF*β*1 and prolonged hypoxia increase the synthesis of endothelin-1 (ET-1) [16]. This vicious cycle gradually causes veno-occlusive dysfunction with the loss of smooth muscle function in the cavernous body [17]. Following apoptosis of smooth muscle cells, cytokines and reactive oxygen species are released from the damaged axons, further aggravating collagen deposition and eventually leading to ED and cavernous tissue fibrosis [18].

### 1.4. Regenerative Medicine in Cavernous Nerve Injury

**Figure 1:**
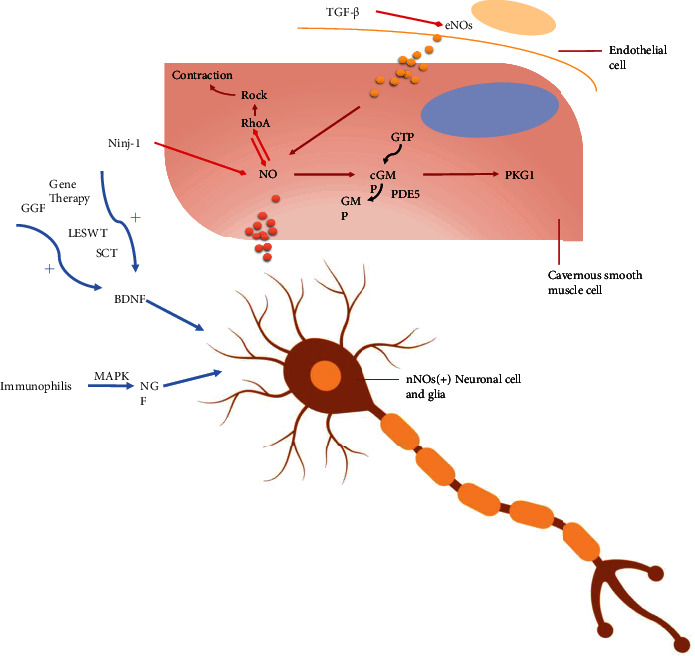
The mechanisms of neurological injury repair. Physiological ED, particularly cavernous nerve damage ED, can be identified as a potential target for different gene therapies. There is an interrelationship between nerve and smooth muscle cells, depicted here, and a simple interrelationship between nerve and smooth muscle cells and between endothelial and smooth muscle cells. All three target cells involved in ED can be targets for different potential gene therapies. The interrelationships between smooth muscle cells and between endothelial and smooth muscle cells are contracted and antagonised, and their diastolic pathways can be enhanced by various gene therapies. Gap junctions between smooth muscle cells allow efficient cell-to-cell signaling. The gene therapy included a large number of trophic factors, not all of which are shown in the figure because they do not or cannot directly influence erectile signaling. Abbreviations: ANG-1: angiopoietin-1; BDNF: brain-derived neurotrophic factor; cGMP: cyclic guanosine monophosphate; eNOS: endothelial nitric oxide synthase; GDNF: glial cell neurotrophic factor; nNOS: neuronal nitric oxide synthase; NO: nitric oxide; PKG1: cGMP-dependent protein kinase G1; RhoA: ras homologous family member type A.

Phosphodiesterase type 5 inhibitors (PDE5i) have been the mainstay of treatment for CNIED for many years, and PDE5i has been used in various types of ED by increasing cGMP levels to counteract cavernous tissue fibrosis. Although they have been shown to be effective in some populations, patients with CNIED are generally complex and PDE5i is extremely dependent on the NO pathway and only works in those with preserved nerves, resulting in poor efficacy [19]. In a study addressing a large number of clinical investigations, Bond et al. noted that patients with CN injury were hypersensitive to sildenafil, with response rates to sildenafil ranging from 10% to 76% in patients after nerve preserving RP (NSRP) and only 0%-15% after nonpreserving RP (NNS RP). And this study found no significant rebound in nNOS-positive nerve density after PDE5i treatment in CNIED patients [16]. In order to more effectively restore EF in patients with CN injury and to address the limitations of clinical treatment with PDE5i, the mechanisms of neurological injury repair have become a focus of research in recent years (Figure 1, Table 1).

## 2. Gene Therapy

### 2.1. Growth Factor (GGF)

Benjamin et al. first found in a rat model of CN injury that nNOS-positive nerve fibres recovered significantly when given brain-derived nerve growth factor (BDNF) or vascular endothelial growth factor (VEGF) injections and that the combination of these neurotrophic factors (NTFs) also promoted the formation of myelin in Schwann cells [20]. In another controlled trial of BDNF and inhibitors of different molecular pathways in rats with CN injury, Bella et al. demonstrated that BDNFs repair CN primarily through the JAK/STAT pathway [21]. NTFs such as glial cell-derived nerve growth factor (GDNF) and neurotrophin 3 (NT-3) have also been widely used in animal models of various types of nerve injury and have been shown to have nerve fibre recovery capacity19, and growth differentiation factor 5 (GDF-5) can also assist in signaling between axons and Schwann cells [22]. In a 15-year trial of CN injury administered by glial growth factor 2 (GGF-2) injection, GGF-2 demonstrated the ability to repair the integrity of CN fibre myelin and promote recovery of erectile function [23].

In contrast, the dominant negative effector cytokine in the pathological process following CN injury is transforming growth factor *β*1 (TGF*β*1). Histone deacetylase (HDAC) hyperactivates the extracellular signal-related kinase (ERK) and phosphatidylinositol 3 kinase (PI3K) pathways via TGF*β*1, which nullifies the deacetylation of HDAC, leading to vascular remodelling and fibrosis [24, 25].

Although NTFs and TGF*β*1, for example, are key in the regulation of neural repair, cytokine targets are broad and nonspecific, and the risk of adverse effects of directly applied related agents is extremely high, and further validation in the ED therapeutic field is lacking. At the same time, these growth factors can be induced by a variety of interventions to circumvent the potential risks of direct application, setting the stage for various CNIED treatment modalities to be investigated.

### 2.2. Nerve Injury-Inducible Protein-1 (Ninj1) and Angiopoietin (Ang)

Ninj1 expression is low in healthy organisms, and in human peripheral blood, Ninj1 is mainly regulated by monocytes. Ifergan et al. identified an important role for Ninj1 in the infiltration of antigen-presenting cells (APCs) into the central nervous system [26]. It was shown that Ninj1 expression was elevated 7 days after CN injury and then returned to baseline levels. In contrast, after blocking Ninj1 expression, inflammatory infiltration of macrophages, dendritic cells, and APCs was effectively reduced, and vascular degeneration and nerve injury were attenuated. In the treated group of the CNIED rat model, local injection of nerve injury-inducible protein-1 antibody (Ninj1-Ab) into the penis induced phosphorylation of NOS, elevated nNOS and eNOS expression, and downregulated Ang1 through upregulation of Ang2 reduced endothelial cell apoptosis, with over 91% restoration of erectile function in the high-dose group, suggesting that Ninj1-Ab has a dual trophic effect on vascular and nerve regeneration [27, 28].

The current research on Ninj1 antibodies is mainly focused on DM neuropathic ED, and no studies have been reported on its direct application to CNIED. Due to its neurotrophic and restorative capacity, it can be considered that Ninj1 antibodies have the potential to become a new treatment modality for CNIED when the research is refined at a later stage.

### 2.3. RhoA/ROCK Pathway

RhoA is a small molecule monomer of the Ras-GTPase family of G proteins with bioregulatory effects. RhoA signaling in the penis activates Rho-associated protein kinase (ROCK), which inactivates myosin light chain phosphatase (MLCP) and regulates cavernous smooth muscle contraction by mediating the Ca^2+^ sensitization pathway, an erection inhibition process that was thought to be regulated independently of NO signaling in earlier studies [29]. However, subsequent studies demonstrated that ROCK expression was upregulated and RhoA/ROCK pathway activity was enhanced after CN injury in rats, while phosphorylation of eNOS was inhibited and NO synthesis was reduced, showing a negative regulation of the NO pathway [30]. Sopko et al. found that activation of the NO/cGMP/PKG pathway also inactivated RhoA in rat vascular smooth muscle cells, demonstrating that NO also negatively regulates the RhoA/ROCK pathway [31]. This complex interaction was recognized in subsequent studies, suggesting that NO and RhoA synergistically maintain vascular homeostasis in the spongiosa. In a rat model of CN injury, smooth muscle diastole, reduced local tissue fibrosis, reduced axonal apoptosis, and signs of regeneration were observed after inhibition of the RhoA/ROCK pathway. The relative expression of ROCK-2 was higher in patients with CN injury [32], but many studies pointing to higher expression of ROCK-1 as more relevant to CNIED are still controversial [33–35]. Hannan et al. used a nonselective ROCK-1 inhibitor (Y-27632) for intracavernosal injection in a rat model of CN injury, and two weeks later, the rats recovered erectile function, significantly upregulated both nNOS and eNOS expression, and effectively inhibited apoptosis of nNOS-positive axons [36]. The nonselective ROCK-1 inhibitor was also shown to restore CN in a subsequent study [37].

Smooth muscle diastole was evident in Y-27632 in human cavernous (postprosthetic implant removal) applications, and the effect was even more pronounced with the combination of vardenafil. Because RhoA/ROCK inhibitors act on a wide range of upstream sites, they have been used in current clinical studies for the treatment of cardiovascular disease. For the treatment of CNIED, Löhn et al. selected the more selective ROCK inhibitor SAR407899 for evaluation, and clinical phase II trials have been completed, but the results have not yet been published [38]. Combined with a large number of experiments in animals with good results, it can be assumed that RhoA/ROCK inhibitors will be a new option for the combination treatment of CNIED.

## 3. Immunophilin

Immunophilin is a specific receptor protein for immunosuppressants and was first recognized in the immune system as the receptor protein that binds cyclosporine, rapamycin, and the novel immunosuppressant tacrolimus (FK506). At the end of the last century, FK506 began to be used in animal models of peripheral nerve injury, and its ability to enhance nerve recovery was demonstrated [39]. Subsequent studies have revealed that proexemptin is also widely present in the central nervous system [40–42]. In 2001, FK506 was used for the first time in a rat model of CN injury and it was found that the lipophilic FK506 selectively acted on damaged nerves and increased the number of unmyelinated synapses in the penis of the rats compared to the blank control [43]. Burnett et al. also demonstrated the proneural regenerative ability of FK506 in subsequent experiments [44]. To circumvent immunosuppression and adverse immunosuppressive toxicity during treatment, the tacrolimus analogues GPI-1046, GPI-1485, and FK1706 were developed [44–46]. Studies have shown that FK1706 potently activates the MAPK pathway of nerve growth factor (NGF) and has significant neurotrophic effects by binding FK506 binding protein-52 (FKBP-52) and phospholipase C (PLC) to block inhibition of the MAPK pathway [47].

Despite mostly positive results in animal studies, in 2010, the American Urological Association published the results of a multicentre randomised controlled phase IV clinical trial showing that patients with CN injury after RP had no significant improvement in erectile function during tacrolimus treatment and two years of follow-up but instead experienced serious adverse effects such as nephrotoxicity and neurotoxicity due to long-term immunosuppression [48]. In another clinical study by this society on the preventive effects of low-dose tacrolimus RP after surgery, FK506 was still not shown to be superior to placebo [49]. Clinical trials of tacrolimus analogues for CNIED have since been halted pending the development of safer and more efficient clinical trials with more selective and specific proimmune agents.

In addition to medical techniques such as PDE5i and maturing prosthetic implants, vacuum negative pressure devices and prostilbestrol combined with extracorporeal injections all play an important role in the adjuvant treatment of CNIED, which are mainly involved in the repair of damaged cavernous oxygenation, smooth muscle antiapoptosis, and antifibrotic changes. Regenerative medicine research focuses on promoting functional nerve repair and regeneration through the endogenous regenerative capacity of damaged tissues, and the available regenerative medicine tools for CNIED include gene therapy, stem cell therapy (SCT), tissue engineering, and low-intensity Extracorporeal Shockwave Therapy (LESWT).

## 4. Stem Cell Therapy (SCT)

SCT is an emerging treatment for CNIED. The use of different tissue-derived stem cells such as adipose tissue-derived stem cells (ADSCs) [50], urogenic stem cells (USCs) [11], bone mesenchymal stem cells (BMSCs) [51], and embryonic stem cells for CNIED has been validated in several studies. The use of stem cells from different tissue sources such as ADSCs, urogenic stem cells (USCs), bone marrow mesenchymal stem cells (BMSCs), and embryonic stem cells in CNIED has been validated in several studies. Studies have shown an increase in endothelial, smooth muscle and neural cell markers, a significant reduction in collagen deposition, and neural cell apoptosis in the cavernous body after stem cell treatment, and therefore, the efficacy of stem cell therapy is widely recognized. The mechanism of promoting repair of cavernous nerve damage is thought to be mainly due to the antiapoptotic effects of paracrine NTFs and other cytokines, but there are still challenges in homing kinetics such as low local retention and in vivo migration metabolism.

Clinical studies with stem cells in CNIED have shown that stem cells have the ability to reverse structural damage and apoptosis in cavernous tissues, thereby reducing patients' dependence on the transient effects of PDE5is and reducing drug resistance [52–54]. Various stem cells have been used in a variety of animal models of ED; the main ones that have been successfully validated include mesenchymal stem cells such as adipose-derived stem cells (ADSC). The first report of using stem cells to treat erectile dysfunction came from Bochinski et al. They differentiated embryonic stem cells taken from rat blastocysts into neural cells by transfecting BDNF and injected them into the large pelvic ganglion of rats, and the treated group showed a significant improvement in erectile function and a significant increase in NF expression [55]. Since this study, a number of preclinical trials have evaluated the efficacy of stem cells in vascular erectile dysfunction, and the results of these studies have shown an improvement in erectile function [56, 57]. Cell binding in animal models of ED in chronic disease states has also been debated by the Knuppe Laboratory of Molecular Urology in San Francisco, as they showed that in animal models of type 2 diabetes and hyperlipidemia, erectile function improved after treatment with ADSCs, but without significant cell binding. Thus, although the mechanism of action of stem cells in the treatment of ED in cavernous nerve injury is becoming clear, there is still controversy about the possible role of stem cells in the treatment of ED without an acute pathogenetic cause. Some of the stem cell studies described above also illustrate an exciting avenue for regenerative medicine between gene therapy and stem cell applications, as stem cells are increasingly being used as vectors to deliver genes to desired tissues [58, 59].

Structural changes seen in the cavernous body after SCT include increases in endothelial and smooth muscle cell markers, increases in neuronal cell markers, decreases in collagen content, and decreases in CN cell apoptosis. Importantly, preclinical studies have confirmed that treatment with intracavernous SCT in the cavernous body is safe for rats and can serve as an appropriate model, thus facilitating further studies. Recent advances in SCT studies have facilitated clinical trials in men after RP. Haahr et al. obtained autologous ADSC by liposuction in 17 patients with refractory ED after RP liposuction [50]. Intracavernosal injection of ADSC into the cavernous body had no significant complications and improved IIEF scores. Similarly, a recent study included 12 patients and used bone marrow-derived cells in post-RP patients to confirm the safety of intraluminal SC injection [60]. Further follow-up and more patients in phase II clinical trials are needed to further evaluate the exciting applications of SCT. It is believed that the combination of stem cell and gene therapy or growth factors will become the standardized treatment of choice for patients with CNIED in the future.

### 4.1. Tissue Engineering

Tissue engineering is widely and maturely used in areas such as burn skin vascular transplantation, artificial bladder replacement, and some of these stem cell therapy applications are also part of the modality. Tissue engineering is currently used in the field of erectile dysfunction treatment mainly for artificially constructed structural scaffolds seeded with cell implants. Kershen et al. were the first to use polyethanol polymers to grow endothelial cells and cavernous smooth muscle cells for implantation in vitro, and the grafts were shown to form vascular tissue in vivo, significantly increasing the maximum ICP in experimental animals [61]. Song et al. used whole penile corpus cavernosum cells (excisional surgical specimens) combined with microarterial perfusion and urethral catheter perfusion protocols to reduce clinical antigenicity, constructed evidence of hybrid decellularised scaffolds, and successfully supported cell reseeding to construct a cavernous sinus vascular network [62]. Developments in materials science and 3D printing technologies have simultaneously provided ideas for alternative tissue engineering treatments for ED. Ji et al. validated a rabbit model of cavernous sinus tissue injury using a 3D printed hydrogel scaffold, and the biodegradable scaffold supported regeneration of cavernous vessels, tissue morphology, and enhanced endothelial pressure elasticity while being low in immunogenicity [63]. While most of the current tissue engineering studies focus on the restoration of cavernous tissue supply and metabolism, some studies have also started to focus on the repair of cavernous nerve injury, with artificial nerve replacement and signaling becoming new topics after the failure of earlier autologous nerve grafting techniques.

### 4.2. Low-Intensity Extracorporeal Shockwave Therapy (LESWT)

Past studies have demonstrated that LESWT can repair endothelial damage and improve tissue blood supply in rat cavernous tissue [64]. Consistent with these findings, LEWT pretreatment of penile tissue prior to radical prostatectomy significantly reduced inflammatory markers in cavernous tissue and attenuated a series of injury complexes resulting from cavernous nerve pathway injury, while LEWST inhibited apoptosis and promoted cell proliferation and microvascular regeneration within the tissue [65]. In addition, LEWST has been found to enhance the homing of circulating EPCs to cavernous tissue, the mechanism of this action is currently unclear, and studies speculate that this may be via the SDF-1/cxcr7 pathway [66]. High-energy shock waves were originally introduced for clinical use in extracorporeal lithotripsy; however, in recent years, low-energy shock waves have also been progressively introduced and practised in the urological field, with one previous study even demonstrating that LESWT can reduce oxidative damage and inflammation in renal tissue caused by high-energy shock wave lithotripsy [67]. There is also growing evidence that the use of LESWT can reduce substantial damage to various organs or tissues (e.g., the heart) and improve the function of the heart muscle [68], joints [69], bladder [70], and penis [71]. This may be due mainly to its ability to activate cell proliferation and to inhibit inflammation and promote neovascularisation [72, 73]. In addition, there were few reported side effects of LESWT application that caused significant damage to normal tissues, which further validates its safety [74]. Nevertheless, the efficacy of LESWT in the treatment of cavernous nerve injury erectile dysfunction needs to be further explored, including the effective energy frequency, pulse width, and pulse interval for nerve repair.

## 5. Conclusion

The cavernous nerve structure is complex and prone to injury, and today, cavernous nerve injury erectile dysfunction has become a nonnegligible clinical problem after RP surgery that requires attention. Its prevalence remains high with the development and promotion of the procedure. For many years, PDE5i has been the first-line treatment modality for CNIED. Although studies have demonstrated the efficacy of these conventional drugs, which are primarily used for vascular erectile dysfunction, cavernous tissue damage is more complex in patients with CNIED who have nerve damage, resulting in poorer efficacy and resistance to these drugs. The discovery and breakthrough of pathways represented by RhoA/ROCK signaling continue to provide CNI-specific targeted targets for alternative treatment modalities to conventional PDE5i interventions.

Despite the lack of adequate clinical trial reports, initial basic experimental exploratory work on these therapies has been completed or utilised in neurogenic pathology in other tissues, bringing hope for urological applications. It is believed that conventional treatments for cavernous nerve injury erectile dysfunction will be severely challenged under current therapeutic thinking and that stem cell-related regenerative medicine techniques will play a greater role.

## Figures and Tables

**Table 1:** EF in patients with CN injury.

Study	Model	Regulation; mode of administration	Time point of evaluation	Functional or molecular outcome
1999 Bavetta S, Hamlyn PJ, et al.	(rat)	FK506	12 weeks	Spared axons in the dorsal column
2001 Sezen SF, Hoke A, et al.	(rat)	FK506	14 days	Enhanced preservation of penile innervation
2002 Kershen RT, Yoo JJ, et al.	(mouse)	Reconstitution of human corpus cavernosum smooth muscle	24 days	Maintenance of the smooth muscle phenotype confirmed
2006 Bella AJ, Lin G, Tantiwongse K, et al.	(rat)	Upregulation of penile iNOS	48 hours	Increased sensitivity of nitrinergic cavernosal tissue
2006 Hayashi N, Minor TX, et al.	(rat)	FK1706	8 weeks	Restored axon shape and staining patterns
2007 Valentine H, Chen Y, et al.	(rat)	FK506	7 days	Improved ICP to CNE
2011 Fricker FR, Lago N, et al.	(mouse)	Ninjurin-1	2 months	NRG1-deficient axons found to regenerate at a slower rate
2011 Ji C, Min F, et al.	(MDSC)	Tissue-engineered corpus cavernosum with muscle-derived stem cells	6 months	Extracted all cellular components while preserving the original collagen fibres
2013 Lasker GF, Pankey EA, et al.	(rat)	Rho-kinase inhibitor azaindole-1	2 hours	Improved ICP to CNE
2013 Yin GN, Kim WJ, et al.	(mouse)	Ninjurin-1	7 days	Improved ICP to CNE
2015 Burnett AL, Sezen SF, et al.	(rat)	GGF2	5 weeks	Schwann cells increased
2015 Cho MC, Park K, et al.	(rat)	Rho-kinase inhibitors	4 weeks	Decreased smooth muscle-to-collagen ratio
2015 Song SH, Park K, et al.	(rat)	Rho-kinase/LIM kinase/Cofilin	4 weeks	Reduced ICP to CNE
2016 Haahr MK, Jensen CH, et al.	Post RP ED men	Autologous adipose-derived regenerative cells	24 months	Recovered erectile function
2017 Campbell JD, Burnett AL.	(rat)	Upregulation of eNOS	21 days	Improved ICP to CNE
2017 Ding Z, Shen X, et al.	(rat)	Rho-associated kinase	—	Improved ICP to CNE
2017 Gao W, He X, et al.	(rat)	FK1706	4 months	Higher S100*β* and GAP43
2017 Uvin P, Albersen M, et al.	Post RP ED men	Rho-kinase inhibitor Y-27632	—	Relaxation of corpus cavernosum in tissue strips
2018 Park J, Cho SY, et al.	(rat)	Inhibiting LIM-kinase2	1 week	Improved ICP to CNE
2020 Yang Q, Chen W, et al.	(rat)	Adipose tissue-derived stem cells and endothelial progenitor cells	1 month	Improved ICP to CNE

## Abbreviations

ED: Erectile dysfunction
CNI: Cavernous nerve injury
CNIED: Cavernous nerve injury erectile dysfunction
EMAS: European Male Ageing Study
nNOS: Neuronal NOS
eNOS: Endothelial NOS
PCa: Prostatic cancer
RP: Radical prostatectomy
EF: Erectile function
GTP: Guanosine 5t-triphosphate
GGF: Growth factor
PKG: Protein kinases
NSRP: Nerve preserving RP
PDE5i: Phosphodiesterase type 5 inhibitors
VEGF: Vascular endothelial growth factor
APCs: Antigen-presenting cells
cGMP: Cyclic guanosine monophosphate
GDNF: Glial cell-derived nerve growth factor
SCT: Stem cell therapy
ADSC: Adipose-derived stem cells
NT-3: Neurotrophin 3
FKBP-52: FK506 binding protein-52
PLC: Phospholipase C
LESWT: Low-intensity extracorporeal shockwave therapy
ET-1: Endothelin-1.
